# Quantitative expression of developmental genes, Pou5f1 (Oct4) and Mest (Peg1), in vitrified mouse embryos

**Published:** 2013-09

**Authors:** Masoumeh Rajabpour-Niknam, Mehdi Totonchi, Maryam Shahhosseini, Ali Farrokhi, Hiva Alipour, Poopak Eftekhari-Yazdi

**Affiliations:** 1 *Department of Embryology, Reproductive Biomedicine Research Center, Royan Institute for Reproductive Biomedicine, ACECR, Tehran, Iran.*; 2 *University of Science and Culture, Tehran, Iran.*; 3 *Department of Genetics, Reproductive Biomedicine Research Center, Royan Institute for Reproductive Biomedicine, ACECR, Tehran, Iran.*; 4 *Department of Stem Cells and Developmental Biology, Cell Science Research Center, Royan Institute for Stem Cell Biology and Technology, ACECR, Tehran, Iran.*

**Keywords:** Mice, Blastocyst, Vitrification, Oct4, Mest

## Abstract

**Background:** Embryo cryopreservation is the process that water is removed from the cell by cryoprotectant materials, and embryos are stored at temperature below zero. This process may affect the viability and developmental potential of embryos.

**Objective: **In this study, the effect of the vitrification cryotop method on the expression level of Oct4 and Mest developmental genes in mouse blastocysts was examined.

**Materials and Methods:** The collected 2-cell embryos of superovulated mouse by oviduct flushing were divided into non-vitrified and vitrified groups. These embryos were cultured to the blastocyst stage directly in the non-vitrified group and in the vitrified group, these embryos were cultured to 4-8 cell embryos, vitrified with cryotop in these stages and after 2-6 months, warmed and cultured to blastocyst embryos. Quantitative expression of two developmental genes, namely Oct4 and Mest, were performed in these groups, using RNA purification and Real-time RT-PCR.

**Results: **Quantitative PCR analysis showed that the expression level of both genes, Oct4 and Mest, was reduced significantly in the vitrified-warmed group relative to the control group (p=0.046 and p=0.001).

**Conclusion:** This study revealed that morphologically normal embryos show a reduced amount of Oct4 and Mest transcripts which indicate that the vitrification method negatively effects the expression level of these two developmental genes.

## Introduction

Vitrification is a cryopreservation protocol, which was first described by Fahy and Rall in 1985. It is a cost-efficient method widely used for the cryopreservation of mammalian embryos (1). During this process cells are frozen into a glassy shapeless solid with no crystals, which is possible with the use of a compound of cryoprotectants and very low temperatures (2). Since ice crystals are not formed in this method, the negative effects of freezing on the embryo are reduced to a minimum (1). 

Vitrification cryotop is a very easy and reliable method which can be used for the cryopreservation of human ova and embryos during all development stages (2). Although in vitro produced and vitrified embryos look morphologically normal, there is no guarantee on their natural development. Recently, there has been a report about the harmful effects of vitrification on an obstetric outcome. In 2010, Wikland *et al* found an increased risk of post-partum haemorrhage after transfer of vitrified blastocysts using cryoloop, which could be speculated that the vitrification process itself somehow affects the trophoectoderm cells in a way that may influence placentation, negatively (3).

A subset of genes within the mammalian genome is expressed from only one of the two alleles depending on father or mother heredity of gene. Such genes which have a parent-of-origin-specific manner of expression are known as “imprinted genes”, and the corresponding epigenetic mechanism is called genomic imprinting (4). 

Almost all imprinted genes identified to date have the roles as regulators of embryonic growth, placental growth or adult metabolism (5). The Mest gene is an imprinted gene which encodes a hydrolase enzyme (6). The Mest cDNA is about 2.5 kb and encodes a polypeptide of 335 amino acids (7). Kaneko-Ishino *et al* have demonstrated that this gene is the imprinted Peg1 gene which is only expressed paternally (8). This gene has been mapped to proximal mouse chromosome 6 and maternal duplication of chromosome 6 causes early embryonic lethality (9). 

In some embryos, the onset of imprinted MEST expression occurs during late preimplantation (10). The PEG1 gene (MEST) is expressed in human placental trophoblast and endothelium, and data from knockout mice show that this gene regulates placental and fetal growth (11). The transcription factor Oct4, also known as Oct3 or Pou5f1, is a member of the POU domain family from the octamer binding proteins (12). The Oct4 gene encodes a protein of 352 amino acids which contains a protected region of 150 amino acids called POU domain. This gene contains five exons which encode a transcript of about 1.5 Kilo base (13). 

The Oct4 has been mapped to mouse chromosome 17 (14). Oct4 can be detected in all cells up to the late blastocyst stage and is gradually eliminated from the trophoectoderm (15). Oct4 also plays a critical role in the development of the embryo; for example, Oct4 null homozygous embryos die around the time of implantation (16). Through its influence on both the transcriptional and post-transcriptional regulators, this gene is able to directly or indirectly affect many of the essential processes of early development such as chromatin remodeling, epigenetic regulation, apoptosis, cell cycle regulation and signaling (17). 

This study has tried to assess the effect of the vitrification cryotop method on the expression of two developmental genes (Mest and Oct4) in mouse preimplantaion embryos.

## Materials and methods

This is an experimental study. All chemicals used in this study were purchased from Sigma Chemical Co. (St. Louis, MO, USA) unless otherwise stated. Similarly, all embryo culture dishes used for the culture of embryos were Falcon-ware (Becton-Dickinson, Bedford, MA, USA). All experiments were approved by the Royan Institute Animal Ethics Committee.

**Study groups**

This study consisted of one control (non-vitrified) and one experiment group (vitrified-warmed). In the control group, 2-cell embryos flushed from the oviduct were allowed to develop to the blastocyst stage (late blastocyst and following stages). In the experiment group, the retrieved 2-cell embryos were vitrified after reaching the 4- to 8-cell stage and kept for 2-6 months in liquid nitrogen. After warming, they were cultured to the blastocyst stage as with the control group. Analyses were performed on blastocysts which were morphologically normal (thin zona pellucida, smooth trophoectoderm, clearly visible blastocyst cavity and well-developed inner cell mass) (18).

**Embryo collection**

Embryos were obtained from 6- to 8-week old female NMRI (Naval Medical Research Institute) mice which were superovulated by an intraperitoneal injection of 7.5 IU/ml pregnant mare’s serum gonadotrophin (PMSG; Intervet Folligon 5000 IU; Holland) followed 48h later by 7.5 IU/ml hCG (Organon 500 IU; Holland). The females were placed overnight with NMRI males of proven fertility, as previously described (19). The 2-cell embryos were collected at 48h after hCG injection by oviduct flushing and a total of ten embryos were placed into the 20 ml culture medium drop [T_6_ medium containing 4 mg/ml bovine serum albumin (BSA)] and incubated at 37ºC in 5% CO_2_ in air. 

**Embryo vitrification**

The entire vitrification process was performed at room temperature. Five to ten normal embryos in the 4- to 8-cell stage (equal-sized blastomeres, <10% fragmentation and clear cytoplasm in all blastomeres) were placed in the equilibration solution (base medium, 7.5% v/v DMSO and 7.5% v/v ethylene glycol) for 7 minutes then in the vitrification solution (base medium, 15% v/v DMSO, 15% v/v ethylene glycol and 0.5M sucrose) for 1 min. Embryos were moved onto a cryotop (Kitazato, Japan) which was immediately submerged in filter-sterilized liquid nitrogen. The embryos were kept in it for a period of 2-6 months. Base medium consisted of Ham’s F10 medium containing 10% human serum albumin (HSA; Octapharma, Switzerland).

**Embryo warming**

The cryotops containing the vitrified embryos were quickly transferred into the first pre-warmed (37^o^C) warming medium (base medium containing 1M sucrose) for 1 min before being moved to the second warming medium (base medium containing 0.5M sucrose) for 3 min at room temperature and finally the third warming medium (base medium containing 0.25M sucrose) for 3 min. The warmed embryos were washed several times in the wash medium (base medium), transferred to T6 (containing 4mg/ml BSA) drops under mineral oil and incubated at 37^o^C, 5% CO_2_ until they reached the blastocyst stage.

**Gene expression**

Reverse transcription-polymerase chain reaction (RT-PCR) was used to assure the expression of the mentioned genes in both the control and experiment groups. Real-time RT-PCR was used to assess the quantitative expression of these gene transcripts. Primers used for both of the above procedures were designed using primer design softwares: Primer3 (Whitehead Institute for Biomedical Research) and perlprimer as listed in table I (20).

**RNA extraction and RT-PCR**

The AllPrep DNA/RNA Micro Kit (Qiagen, USA) was used to extract RNA from blastocyst embryos. First strand cDNA was prepared from 1mg extracted RNA in a total volume of 20ml using the Revert Aid^TM^ H-Minus First Strand cDNA Synthesis Kit (Fermentas, Germany). RT minus sample was prepared for evaluation of sample DNA contamination in cDNAs. PCR was performed for 34 cycles including denaturation at 94^o^C for 40s, annealing at 60^o^C for 40s and extension at 72^o^C for 40s. β-tubulin was used as the housekeeping gene. RT minus reaction and no template control (NTC) were performed for evaluation of DNA contamination and PCR reagent contamination, respectively. In NTC, RNase-free water was added instead of the cDNA sample. PCR products were loaded onto 1.8% agarose gels and observed by ethidium bromide (0.5 mg/ml) staining and a Transilluminator device (UVidoc, UK). 

**Real-time RT-PCR**

For the assessment of quantitative gene expression, real-time RT-PCR was performed using Rotor-Gene^TM^ 6000 (Corbett Life Science, Australia). The reactions contained 12.5ml SYBR^®^ Premix Ex Taq^TM^ II (Takara Bio. Inc., Japan), 1 ml each of the forward and reverse primer (5 pmol/ml) and 2 ml cDNA samples in a 25 ml reaction. After an initial denaturation step at 95^o^C for 10 min, amplification was performed with 40 cycles of denaturation at 95^o^C for 10s, annealing at 60^o^C for 30s and extension at 72^o^C for 30s. Amplification was followed by a melting curve analysis to confirm PCR product specificity according to the manufacturer^’^s instructions and finally PCR product size was assessed by gel electrophoresis.

For each group, the samples were examined in three independent experiments. These samples were run in duplicate and the mean C_t_-value of each duplicate was used for further calculations. The reactions of RT minus samples, no template controls and studied samples were performed synchronously. The output data which was obtained by Rotor-Gene 6000 analysis software (version 1.7) (Corbett Life Science, Australia), were transferred to REST-MCS (Relative Expression Software Tool-Multiple Condition Solver, 2008, V2.0.7) for quantitation of gene expression. 

The mathematical model used in this software is based on the correction for exact PCR efficiencies and the mean crossing point (CP) deviation between sample and control group, as follows:

Ratio=(*E*_target_)^ΔCt ^target (Mean control- Mean sample)/(*E*_ref_)^ΔCt^ref (Mean control- Mean sample)

b-actin as a reference gene was used for normalization of data.

**Statistical analysis**

The expression ratio results of the investigated transcripts were tested for significance by Pair Wise Fixed Reallocation Randomization Test^©^ (21) and plotted using standard error (SE) estimation via a complex Taylor algorithm using the REST-MCS Software (http://rest.gene-quantification.info/). The percentage of normal blastocysts in the vitrified-warmed group compared to the control group was tested for significance by the Chi-square test using the statistical package for the social studies (SPSS 11.5; Chicago, IL, USA) software. The significance was assigned at p<0.05.

## Results

**Embryo development**

After embryos developed to the blastocyst stage, those embryos which had normal morphology were selected for molecular studies. In table II the amount of normal blastocyst and percentage of transformation of normal 4- to 8-cell embryos into the late blastocyst stage and following stages are presented. This scale in the control and experiment groups was reported as 86.8% and 72.3%, respectively, which was a significant decrease (p=0.00) in the experiment group in comparison to the control group.

**Oct4 and Mest genes expression in the studied groups**

**Figure 1:**
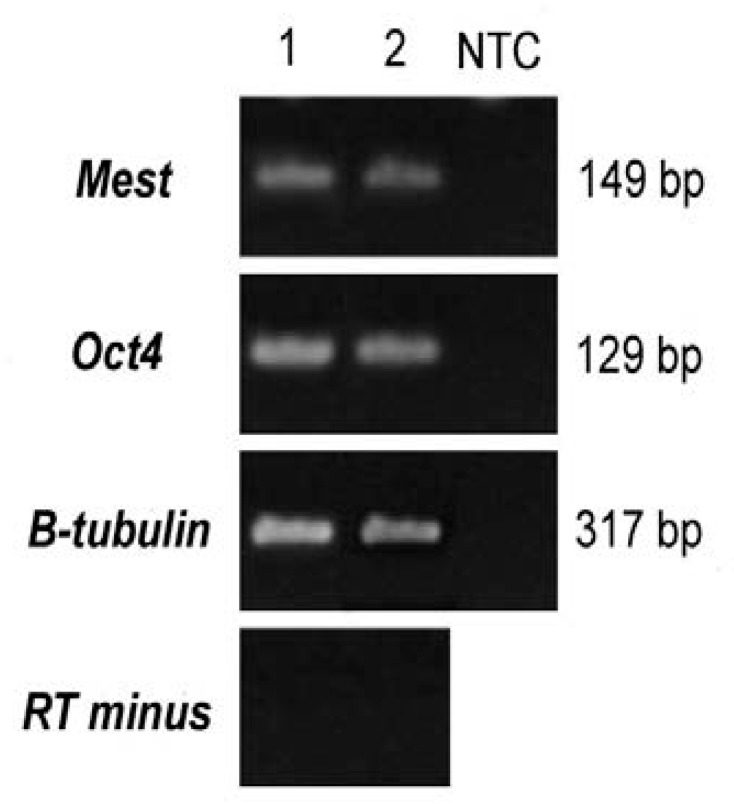
Evaluation of PCR products on 1.8% agarose gel. Column 1: The vitrified group. Column 2: The control group. **NTC column: the same no template control which is performed for evaluation of PCR reagents contamination. RT minus reaction was performed for evaluation of DNA contamination and b-tubulin was used as a housekeeping gene

To confirm the expression of the mentioned genes in both the experiment and control groups, RT-PCR was performed at the RNA level. The results of this procedure have been shown in Figure 1 after agarose gel electrophoresis which revealed expressions of both genes in both groups. For evaluation of quantitative expression of these genes, real-time RT-PCR was performed. 

**Figure 2:**
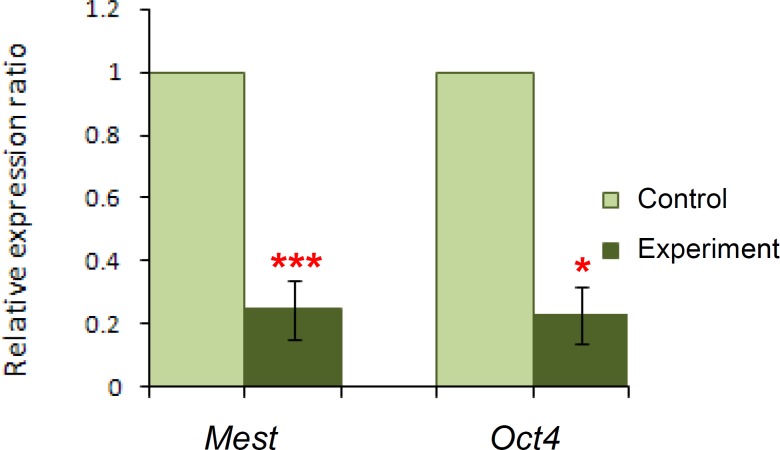
Quantitative expression of Oct4 and Mest gene transcripts in mouse blastocysts of the control and experiment groups. The control group was assigned a value of 1. (*) p<0.05, (***) p=0.001 by the Pair Wise Fixed Reallocation Randomisation Test©.

Embryos at the late blastocyst and later stages were randomly selected and divided into three separate pools (each pool included 35-40 blastocysts) as three experimental replications. The results revealed that the expression levels of Oct4 and Mest genes have a significant decrease (p=0.046 and p=0.001, respectively) in the experiment group compared to the control group (Figure 2).

**Table I:** The primers used in RT-PCR and real-time RT-PCR

**Genbank code**	**Product size(bp)**	**Sequence of primers (5'-3')**	**Gene name**
NM_008590	149	F: CATCGTCCTCTCCTTCTCC	Mest (Peg1)
		R: GTCCCACAGCTCACTCTC	
NM_013633	129	F: GAACTAGCATTGAGAACCGT	Oct4 (Pou5f1)
		R: CATACTCGAACCACATCCTTC	
NM_007393	67	F: CAACGAGCGGTTCCGATG	b-actin
		R: GCCACAGGATTCCATACCCA	

**Table II:** The percentage and amount of normal blastocysts produced from cultures of vitrified and control 4-8 cell mouse embryos

**Groups**	**Normal 4-8 cell embryos**	**Normal blastocyst embryos (%)**
Control	190	165 (86.8) ^a^
Experiment	191	138 (72.3) ^b^

Values within columns with different letters are significantly different (a compared b) (p=0.00) by Chi-Square Test.

## Discussion

Nowadays the vitrification method is very popular compared to slow cooling because it is simple, inexpensive and faster than slow cooling, and protects embryos from damage caused by cooling (22). In 2008, Amr Kader *et al* found that the DNA integrity of vitrified embryos is better than slow freezing with propandiol (23). In 2007, Al-hasani *et al* reported a high survival rate (89%) for human zygotes vitrified using Cryotop (24). The results of this study also showed a comparatively high survival rate for the vitrified-warmed embryos by the cryotop method.

ART methods attempt to increase the safety of mother and embryo. Routinely, after warming, the embryos were transferred which have a normal morphology, high survival rate and successful development to the late blastocyst stage and does not significantly differ from normal control embryos, but there are very few studies on follow-up of children after transfer of vitrified oocytes and embryos. Also Wikland *et al* observed a higher rate of post-partum haemorrhage in the vitrified blastocyst group as compared with the fresh group that the underling mechanism for this effect is not clear (3).

The results of the effect of modified droplet vitrification on apoptotic genes such as Bax, Bcl2 and P53 show that the gene expression patterns in mouse vitrified zygotes and 2-cell embryos which developed to the blastocyst stage have a significant difference (p<0.05) with control embryos (25). According to the results of one study in 2012, vitrification of in vitro- produced bovine blastocysts causes significant up-regulation of genes that are involved in stress responses (26). In 2012, Sudano *et al* showed vitrification alters the expression profile of the genes involved in apoptosis (PRDX2), heat shock (HSPA5), maternal recognition of pregnancy (IFNT2 and PAG2), and cell differentiation and placenta formation (KRT18) that can be related with embryo postcryopreservation survival capacity (27).

Considering the effect of vitrification on expression of some genes, it is possible that vitrification changes some of the genes involved in normal development and reduces their expression, which results in the developmental arrest of the embryos in the next stages of development. In this study, the expression of the two mentioned developmental genes showed a significant decrease. In this study, the effect of vitrification cryotop of 4- to 8-cell embryos on developmental genes (Oct4 and Mest) expression level was experimented. The novelty of this study is the effect of vitrification method in this development stage on Mest expression level in blastocysts.

The appropriate expression of imprinted genes is essential for natural development in mice and the loss of imprinting has been associated with developmental abnormalities and carcinogenesis in the human (28). The localization and timing of PEG1 (MEST) mRNA expression in the human placenta suggest that this gene has a role in placental and decidual angiogenesis (6). Genetic experiments have shown that the mouse orthologue, Peg1, controls prenatal growth. This was discovered by the observation of growth retardation of the fetus and the placenta in Peg1-deficient concept uses (29).

The targeted suppression of the Mest gene in mice results in the inhibition of somatic growth and suggests that these genes result in growth progress and thus the loss of its postnatal expression can contribute to the reduction of growth speed. The loss of Mest results in a decreased body size at birth or earlier which points to the retardation of embryonic growth and in phenotype of abnormal size placenta was observed loss of Mest (30). 

These results and the determination of the important role of Mest in embryo development, and reduction of this gene expression by embryo vitrification in this study suggest that this reduction could be associated with the defects of embryo development. Oct4 plays a critical role in embryo development. This gene has an essential role in maintaining pluripotency of cells of the inner cell mass (ICM) and embryonic stem cells. Oct4 null homozygous embryos die around the time of implantation (16). 

Oct4-deficient embryos survive through the morula stage, but cannot form an ICM (31). These embryos have peri-implantation lethality (16). In this study, the Oct4 gene expression showed a significant decrease (p<0.05) in the experiment group compared to the control group embryos. This decrease in the expression of an important developmental gene can negatively affect development. The Oct4-deficient embryos which have the maternal Oct4 proteins can develop to the blastocyst stage but their ICM is not pluripotent and will die after implantation (31). Maternal Oct4 RNA and proteins are found in the zygote until the 2-cell stage. However, zygotic Oct4 gene expression starts at the 4- to 8-cell stage (32). Despite rapid degradation of maternal Oct4 transcripts starting at the 2-cell stage, maternal and embryonic Oct4 transcripts may transiently exist together (17, 31). 

In one study, it has been revealed that treatment of EC (embryonic carcinoma) cells with RA (retinoic acid) or DMSO (dimethyl sulphoxide) induces differentiation and represses Oct4 mRNA and protein expression (33, 34). In 2011, Zhu *et al* compared the clinical outcome of fresh versus vitrified-warmed human blastocyst transfer (BT) cycles and the vitrified-warmed with the cryotop system BT groups resulted in statistically significantly higher CPR (clinical pregnancy rate) and IP (implantation rate) compared with fresh groups (35). In their study, has not been done any molecular and follow-up study of children after transfer of vitrified embryos, maybe these studies show the different effects of vitrification on development.

In 2012, Zhao *et al* studied the effect of vitrification on the expression levels of pluripotency and differentiation genes (Oct4, Nanog, Cdx2 and Hand1) using Real- time RT-PCR in mouse blastocysts and found the expression levels of Cdx2 and Hand1 were not significantly different in fresh and vitrified blastocysts and the expression of Oct4 and Nanog were increased (36). Their study reveals vitrification has the effect on expression level of Oct4 gene.

Also in 2011, Desai *et al* tested the feasibility of cryopreserving ICM cells using vitrification on both the cryoloop and HSV straw. In their study ICMs were tested for expression of Sox2 and Oct4 using immunoflouresecent staining and they found Oct4 expression increased with time in culture (37). This study showed both Oct4 and Mest gene expression in the late blastocyst stage of vitrified and warmed 4- to 8-cell embryos had a significant decrease compared to control group embryos.

In three above studies, the embryos were vitrified in blastocyst stage but we vitrified them in cleavage stage, perhaps these different results be related to the stage of the vitrified embryo that need more studies. In all these studies, vitrification and molecular assessments have been done in the same stage (blastocyst) but in this study, molecular assessment was done about 36-40h after warming of 4- to 8-cell vitrified embryos. Perhaps this is the cause of the observed different results. As zygotic Oct4 gene expression is started at the 4- to 8-cell stage, maybe vitrification of embryos in after stages affects Oct4 gene expression, differently (32). 

Thus, maybe the cause of the observed problems after transfer of vitrified embryos in our clinic (data not published yet) and other study be the loss of these two important developmental genes during the freeze process and in the successful cases of birth, the effects of the loss of transcripts might have been compensated by replacement with one or more peer transcripts (3). 

The cause of successful development of these embryos to late blastocyst stage despite the decrease of two important developmental genes expression might be the presence of the protein derived from the maternal mRNA which was inherited trough the oocyte and has managed to keep its effect throughout the pre-implantation stages. This reduction of zygotic gene expression affects the later post-implantation stages which show the need for further studies to assess the protein derived from the maternal mRNA in the pre and post implantation embryos.

Considering the importance of these gene expressions on changes in development, the effect of the decrease in these gene expressions in the mentioned freezing process cannot be overlooked. Several factors can affect gene expression and perhaps cryopreservation results in a decrease of the expression of the mentioned genes by affecting epigenetic factors such as DNA methylation and histone modifications. In order to reduce the negative effect of cryopreservation and improve embryo development, further study and research is still needed to find the exact cause of this decrease of expression and the stage which cryopreservation negatively affects. 

## Conclusion

This study showed that vitrification with cryotop has a negative effect in the expression levels of the developmental genes, Oct4 and Mest, in mouse pre-implantation embryos. This determines that the normal morphology of an embryo cannot be a scale of successful embryo development during implantation stages and thereafter, and it requires further study at the molecular level.
